# Regulation of Mitochondrial Dynamics by Proteolytic Processing and Protein Turnover

**DOI:** 10.3390/antiox7010015

**Published:** 2018-01-17

**Authors:** Sumaira Ali, Gavin P. McStay

**Affiliations:** Department of Life Sciences, New York Institute of Technology, Northern Boulevard, Old Westbury, NY 11568, USA; sali51@nyit.edu

**Keywords:** mitochondria, proteolysis, protein half-life, ubiquitin

## Abstract

The mitochondrial network is a dynamic organization within eukaryotic cells that participates in a variety of essential cellular processes, such as adenosine triphosphate (ATP) synthesis, central metabolism, apoptosis and inflammation. The mitochondrial network is balanced between rates of fusion and fission that respond to pathophysiologic signals to coordinate appropriate mitochondrial processes. Mitochondrial fusion and fission are regulated by proteins that either reside in or translocate to the inner or outer mitochondrial membranes or are soluble in the inter-membrane space. Mitochondrial fission and fusion are performed by guanosine triphosphatases (GTPases) on the outer and inner mitochondrial membranes with the assistance of other mitochondrial proteins. Due to the essential nature of mitochondrial function for cellular homeostasis, regulation of mitochondrial dynamics is under strict control. Some of the mechanisms used to regulate the function of these proteins are post-translational proteolysis and/or turnover, and this review will discuss these mechanisms required for correct mitochondrial network organization.

## 1. Introduction

Mitochondria are the power houses of eukaryotic cells, generating chemical energy in the form of adenosine triphosphate (ATP) by the oxidative phosphorylation (OXPHOS) system. Mitochondria are also important for the normal functioning of the cells as they regulate several crucial activities such as differentiation, cell cycle, intracellular signaling and cell death [1]. Mitochondria are unique because of their autonomous DNA (mtDNA), which encodes for proteins required for ATP synthesis. Therefore, the maintenance of mtDNA is important for normal mitochondrial function and for the diversity of the mitochondrial genome [2]. Mitochondria form elongated tubules that continually divide and fuse to form a complex, interconnected and highly dynamic network inside of cells. These dynamic processes not only regulate mitochondrial function but also mitochondrial shape, content exchange and mitochondrial communication with the cytoskeleton [3]. Due to the involvement of mitochondria in a large spectrum of cellular functions, these organelles play a key role in mediating cellular homeostasis. As a result, a healthy population of mitochondria is critical for cell survival. 

Mitochondria constantly undergo fission and fusion to adapt to changes in their ever-changing physiological environment. Both fusion and fission occur in a constant and balanced manner in order to maintain the morphology of the mitochondria and regulate the cellular ATP levels. Mitochondrial fission and fusion are highly regulated by post-translational modification [4]. Mitochondrial fusion produces tubular mitochondria for exchanging material between mitochondria and equal distribution of metabolites. Fusion is mediated by three key regulatory protein fusion proteins Mitofusin1 (MFN1) and MFN2 and optic atrophy 1 (OPA1). The dynamin-related GTPases—MFN1 and MFN2—are responsible for the fusion of outer mitochondrial membranes (OMMs) and form homo-oligomeric and hetero-oligomeric complexes [5,6]. MFN2 is also present in the endoplasmic reticulum, controlling its morphology and facilitating mitochondrial calcium influx from the endoplasmic reticulum [7]. Inner mitochondrial membrane (IMM) fusion is mediated by OPA1, also a dynamin-related GTPase protein, which is associated with different functions such as maintenance of the respiratory chain, IMM potential, mtDNA and control of apoptosis [8]. Its downregulation leads to aberrant cristae remodeling and the release of cytochrome c. YME1L (yeast mitochondria escape-1 like) protease cleaves OPA1 into its long and short isoform. L-OPA1 is integral in the IMM, and S-OPA1 is located in the intermembrane space (IMS) [9]. When mitochondria are depolarized by mitochondrial uncoupling, L-OPA1 is further cleaved by the inducible protease OMA1 (overlapping activity with matrix adenosine triphosphatases asscoaited with various activities protease-1). As a result, mitochondrial fragmentation occurs by preventing mitochondrial fusion [9,10].

Mitochondrial fission not only creates new mitochondria but also allows the segregation of damaged mitochondria and enhanced distribution of mitochondria along cytoskeletal tracks. During fission, the dynamin-related protein (DRP1), which is also a large GTPase, is recruited from the cytosol onto the OMM to constrict mitochondria, resulting in the eventual division of mitochondria [11,12]. In mammalian cells, DRP1 interacts with four mitochondrial receptors proteins: Fis1, mitochondrial fission factor (Mff), mitochondrial dynamics proteins of 49 kDa (MiD49) and 51 kDa (MiD51) [13,14]. The interaction between Fis1 and DRP1 does not have a significant role in regulating mitochondrial fission whereas the interaction of DRP1 with three other receptor proteins plays important roles for fission. Mff helps in the assembly of Drp1 and MID49 and may regulate the DRP1 and maintain its inactive state until fission is required [15]. The reversible phosphorylation of DRP1 by cyclic AMP-dependent protein kinase (PKA) and dephosphorylation by the phosphatase calcineurin results in the recruitment of DRP1 to the mitochondria and promotes mitochondrial fission [9,16].

Beyond fusion and fission, mitochondrial mobility through the cytoskeleton is critically important for the cellular distribution and turnover of mitochondria. In mammalian cells, mitochondria use kinesin/dynein motors to move along the microtubules: kinesin motor towards the plus end and dynein motor towards the minus end of microtubules [17]. The attachment between the mitochondria and microtubules is regulated by the interaction between OMM proteins Miro1 and Miro2 and adaptor protein Milton. Interestingly, both MFN1 and MFN2 interact with Miro and Milton [18]. It has been demonstrated that defects in both fusion and fission decrease mitochondrial mobility and as a result affects mitochondrial morphology [19]. However, the mechanism of interaction between mitochondrial transport and fusion-fission machinery is unclear.

When mitochondrial dynamics are disturbed, cellular dysfunction occurs. Mitochondrial turnover is therefore an integral part of quality control, in which dysfunctional mitochondria are selectively eliminated through mitophagy [20,21]. A healthy mitochondrial population requires a controlled balance between mitophagy and mitochondrial biogenesis. Excessive mitophagy can result in bioenergetic failure [22].

The pathway of mitophagy depends on ubiquitylation, targeting the autophagosome via ubiquitin and microtubule associated protein light chain 3α (LC3)-binding adaptor protein, and the fusion of autophagosome with lysosomes [23]. Mitophagy activated by cellular stress triggers depolarization of the OMM, which results in stabilization of the serine/threonine kinase phosphatase and tensin homolog (PTEN)-induced putative kinase 1 (PINK1) on the OMM and recruitment of the E3 ubiquitin ligase Parkin [24,25]. The interchange between PINK1 and Parkin is a crucial step in mediating the clearance of dysfunctional mitochondrial [26,27]. Parkin-independent mitophagic mechanisms or mitochondrial spheroid formation related to mitochondrial quality control also have been suggested [28]. However, more studies are need to understand the importance of these mechanisms in mitochondrial turnover.

Diseases associated with defective mitochondrial dynamics often manifest in the nervous system and occasionally in muscle. The most commonly known diseases of this type are Charcot–Marie tooth disease (CMTD) and dominant optic atrophy. CMTD is a family of autosomal dominant diseases that result in peripheral neuropathy due to the inability to maintain axonal function. Specific mutations in MFN2 give rise to different sub-groups of the disease that can be early or late onset. Mutations in VCP/p97 are also associated with a sub-group of CMTD. Dominant optic atrophy is a disease resulting in degeneration of retinal ganglion cells. Mutations in OPA1 are responsible for the manifestation of disease, with haploinsufficiency as the mechanism. A subgroup of Optic Atrophy is caused by mutations in the mitochondrial AAA-ATPase protease YME1L [29]. Another disease resulting from defective mitochondrial dynamics are the Encephalopathies due to defective Mitochondrial and Peroxisomal Fission (EMPF). These arise due to mutations in a number of mitochondrial dynamics proteins including DRP1 (EMPF1) and Mff (EMPF2) that are both involved in mitochondrial fission [30,31]. Another disease with a strong relationship to defective mitochondrial dynamics is Parkinson’s Disease. This is a neurodegenerative disease that affects the substantia nigra causing these cells to die and an individual to lose motor control skills. A small percentage of Parkinson’s Disease cases are due to mutations in the Parkin gene and are inherited in an autosomal recessive manner [32]. SLC25A46 and MFN2 are associated with Hereditary Motor and Sensory Neuropathy (HMSN) [33,34] while HUWE1 is associated with X-linked syndromic mental retardation, Turner type (XMRT) [35].

Mitochondrial dynamics are also involved in pathologies associated with high reactive oxygen species (ROS), especially in ischemia-reperfusion injury of the heart. Increasing mitochondrial fusion results in protection against tissue damage resulting from the ischemia-reperfusion episode. Mitochondrial fusion inhibits activation of the mitochondrial permeability transition: an IMM permeabilization event that is associated with necrosis as demonstrated in rat heart and heart cell lines [36]. Mitochondrial fusion and inhibition of fission are associated with lower production of ROS [37] most likely through the maintenance of balanced amounts of ETC components. Protection against opening of the mitochondrial permeability transition can be through increased expression of fusion-promoting proteins, loss of fission-promoting protein expression and inhibition of fission protein function using small molecule inhibitors. It has also been demonstrated that DRP1 can undergo phosphorylation via calmodulin-dependent kinase II (CaMKII) which promotes the activation of the mitochondrial permeability transition under conditions of chronic β-adrenergic receptor stimulation in myocytes [38]. Fragmentation also causes the accumulation of calcium in mitochondria which is a common activator of the mitochondrial permeability transition, particularly in the case of pro-fission proteins such as DRP1 and Fis1 [39]. 

Due to the importance of mitochondrial dynamics in maintaining cellular homeostasis, the regulation of expression of mitochondrial dynamics proteins must be carefully controlled. Protein abundance can be controlled by increases in gene expression, but also via post-translational mechanisms, such as proteolysis and protein stability and turnover. This review will focus on these two types of post-translational regulation of mitochondrial dynamics proteins. 

## 2. Links between Protein Turnover and Mitochondrial Function

Proper mitochondrial function depends on effective quality control of this organelle. Defects in mitochondrial quality control leads to aberrant mitochondrial structure or complete mitochondrial dysfunction. The quality control of mitochondria is mediated by turnover of mitochondria by mitophagy or mitochondrial proteins by the ubiquitin protease system (UPS) or intra-mitochondrial proteolytic systems. Polyubiquitylation of proteins signals for destruction by the UPS, which can occur due to loss of protein structure or function or as part of regulation of signal transduction pathways. In human cells, immunocapture of ubiquitin-tagged and associated proteins revealed that 38% had a mitochondrial localization [40]. Ubiquitin tagging does not only signal for protein degradation but is also used in signal transduction pathways; therefore, the relative amount of proteins targeted for UPS-dependent degradation is lower. Ubiquitin can modify target proteins by using specific lysine residues to form an isopeptide bond. Polymerization of the ubiquitin chain occurs by further addition of ubiquitin monomers onto specific lysine residues on the ubiquitin molecule, most commonly K48, K63, but also K11 and K6 [41]. These latter three modifications have been shown to be enriched on mitochondria after depolarization of the IMM, a consequence of mitochondrial dysfunction. These specific ubiquitin chains can signal to activate mitophagy to remove dysfunctional mitochondria. In the budding yeast, *Saccharomyces cerevisiae*, the UPS is required to maintain correct mitochondrial function under normal homeostatic conditions. When there are defects in the UPS, mitochondrial defects are observed by complete deletion of the SCF E3 ligase complex that ubiquitylates specific proteins, core proteasomal subunits responsible for proteasomal degradation, ubiquitin activating proteins and ubiquitin recognizing proteins [42]. These observations indicate that a constant turnover of mitochondrial proteins is required during standard fermentative growth conditions in yeast. Similar phenomena occur in mammalian models of disease when proteasomal function is inhibited or dysfunctional due to genetic alterations. Neurodegenerative diseases often display proteasomal defects due to the accumulation of neurotoxic molecules such as alpha-synuclein, beta-amyloid or mutant huntingtin which can act as inhibitors of proteasome activity or by overwhelming proteasome activity [43]. Proteasomal involvement in regulation of mitochondrial function is also demonstrated by a number of proteasome components and ubiquitin E3 ligases that associate on the surface of the OMM, such as IBRDC2 (in between ring fingers domain containing 2), FBXW7 (F-box/WD repeat containing protein 7), FBXO7 (F-box only containing protein 7), RFN185 in humans and Rsp5 and Dma1 in budding yeast (see references in [44]. Ubiquitylation of OMM proteins that expose domains and loops to the cytosol can result in one of two outcomes. Ubiquitylated proteins recruit adaptor proteins that then recruit ATPases to extract these proteins from the OMM or as a platform for the initiation of mitophagy. The decision between individual protein extraction or mitophagy is most likely dependent on the number of ubiquitylated proteins on the OMM. Mitochondria-derived vesicles are a recently described mechanism of mitochondrial quality control that target mitochondrial lipids and proteins to other membrane bound compartments such as the peroxisome, endosome and multi-vesicular bodies. These membrane structures are formed with the involvement of the PINK1 and Parkin proteins that act to ubiquitylate OMM surface proteins [45]. This close connection between the UPS and mitochondria indicates the importance of mitochondrial proteins and function to overall cellular physiology. 

The action of UPS-dependent protein turnover in part determines protein stability, which can be measured by determining half-life and indicates the rate of protein loss regardless of mechanism of degradation. Three large scale studies have determined the half-lives of proteins in human cell lines and in budding yeast. Half-lives in human cells varied from 45 min to 22.5 h in 100 proteins. Yellow fluorescent protein-tagged proteins were followed and protein half-lives were determined by loss of bleached protein. Protein half-life determined by this method increased after treatments such as chemotherapeutic agents or inhibitors of transcription, especially for long-lived proteins. In budding yeast, two different approaches were used that resulted in conflicting half-lives for each protein. Following epitope-tagged proteins in yeast treated with cycloheximide gave an average half-life of ~43 min, with some as short as 4 min grown in complete media with glucose, while a proteomic approach, using a heavy isotope of lysine as a pulse, was diluted with non-radiolabelled lysine and displayed much longer half-lives, with a mean of 8.8 h with a cell doubling time of 2.5 h in glucose and synthetic media (Table 1) [46,47,48,49]. These studies indicate that there is selectivity of protein turnover as different proteins have different half-lives. This could be due to specific motifs for turnover in proteins, interactions between proteins, signals activating protein turnover or dilution during cell division.

## 3. Inner Mitochondrial Membrane Fission and Fusion

**Figure 1:**
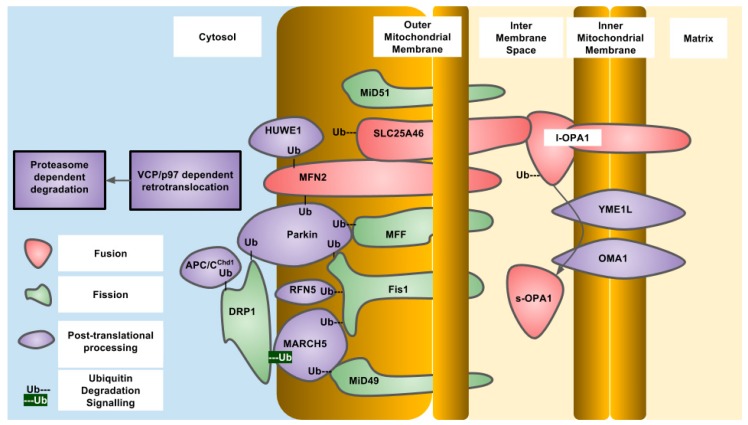
Essential components of human mitochondrial dynamics indicating post-translational ubiquitylation and proteolysis, protein localization and known modifying enzymes.

**Figure 2:**
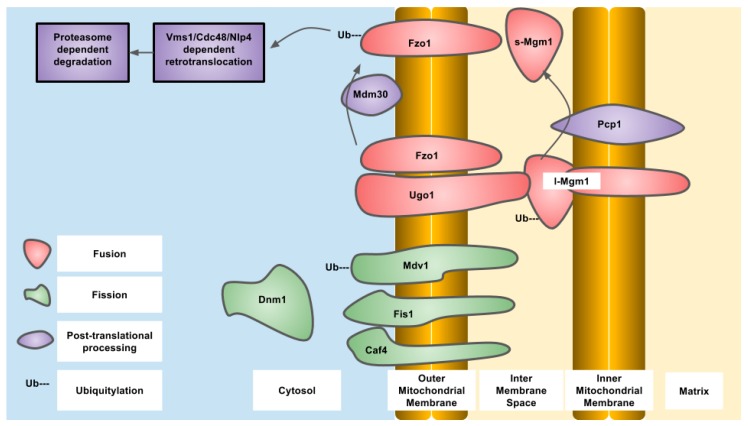
Essential components of mitochondrial dynamics in Saccharomyces cerevisiae, indicating ubiquitylation and proteolysis, protein localization and known modifying enzymes.

OPA1 is an IMM-targeted GTPase involved in fusion of the IMM as well as cristae organization that can also localize to the IMS. The different localizations are due to differential splicing as well as proteolytic processing. OPA1 is proteolytically processed by OMA1, an IMM-resident zinc metallopeptidase, and YME1L, an IMM-resident ATP-dependent metalloprotease. The protease sites are not present in all of the eight different splice variants that exist in humans. The YME1L cleavage site is encoded in exon 5b which is not present in all OPA1 isoforms. Constitutive proteolytic processing by YME1L and/or OMA1 generates a balance of short and long isoforms that are released into the IMS or tethered to the IMM respectively. Upon alterations to mitochondrial physiology, such as loss of mitochondrial membrane potential, ATP depletion or induction of apoptosis, OPA1 is further proteolytically processed by OMA1 to generate the short isoforms of OPA1 that are released into the IMS and do not support mitochondrial fusion, resulting in overall mitochondrial fragmentation. This regulation of OPA1 allows for alterations in the mitochondrial network through post-translational mechanisms that are more rapid than changes in gene expression. Constitutive proteolytic cleavage of OPA1 by OMA1 occurs to balance the rates of mitochondrial fission and fusion to maintain mitochondrial function. The OMA1 cleavage site in OPA1 is C-terminal to alanine at residue 195 and generates short OPA1 isoforms that are not capable of mitochondrial fusion. The S2 site in OPA1 is cleaved by YME1L between the residues 217 and 223 (LQQQIQE) [50]. Under stressed conditions, OMA1 induces cleavage of OPA1 to generate short isoforms. To terminate this signal, OMA1 undergoes autoproteolytic cleavage and is degraded eventually, allowing the long isoforms to accumulate and allow mitochondrial fusion to occur again [51,52]. In the absence of OMA1, the short isoforms of OPA1 cannot be generated, and this results in a fragmented mitochondrial network. Reconstitution of different OPA1 isoforms into OPA1 deficient mouse embryonic fibroblasts demonstrated that both the long and short forms of OPA1 are required to restore a balance of mitochondrial dynamics [53]. OMA1 was first described in yeast as overlapping activity with m-AAA protease, but is not a functional homolog of the human OMA1. Human OMA1 does not rescue a OMA1 deficient yeast strain from respiratory deficiency when also deleted with YME1, the YME1L homolog. The activation and autocatalytic degradation of the human OMA1 expressed in yeast was also induced by loss of mitochondrial membrane potential indicating a domain present in human OMA1 that is sensitive to mitochondrial membrane potential. The amino-terminal domain of human OMA1 is much longer than that of yeast and may contain this domain [51]. Yeast OMA1 still undergoes autoproteolysis after stress induction that is dependent on a carboxy terminal domain involved in stabilization of a homo-oligomeric complex [54]. In yeast, the OPA1 homolog is MGM1 (mitochondrial genome maintenance 1) which also undergoes proteolytic processing to generate two isoforms—one short and one long. The long isoform also has a trans-membrane domain and is tethered to the IMM, while the short isoform is soluble in the IMS. MGM1 is proteolytically processed to generate the short isoform by the PCP1 (processing of cytochrome c peroxidase 1) IMM protease and not OMA1. Similar to OPA1, both MGM1 isoforms are required for a balance of mitochondrial dynamics. PCP1 is homologous to serine proteases, such as rhomboid found in drosophila [55]. The phenotypic consequences of PCP1 deletion seem to be entirely due to lack of MGM1 processing and generation of the short isoform of MGM1. When only short MGM1 is introduced into PCP1 deletion, strains of yeast mitochondrial morphology are partially restored and prevent the loss of mitochondrial DNA caused by defective mitochondrial fusion. In yeast, the balance between long and short forms of MGM1 is also regulated by PSD1, (phosphatidylserine decarboxylase 1) in the IMM that produces phosphatidylethanolamine, indicating regulation of MGM1 processing by mitochondrial lipid composition and indicating the activity of PCP1 is regulated by lipid composition [56] (Figure 1 and Figure 2).

A less well characterized protein, MTP18 (mitochondrial protein of 18 kilodaltons), is a fission factor embedded in the IMM that is conserved in metazoans [57]. Genetic deletion of this protein results in hyperfused mitochondria and overexpression results in excessive mitochondrial fission. MTP18 protein expression is dependent on Phosphatidylinositol 3-kinase activity, and inhibition of this pathway results in loss of MTP18. After serum withdrawal or inhibition of PI3K (phosphatidylinositol 3-kinase) by the small molecule inhibitor LY294002, MTP18 protein decreases in expression by 50% in approximately 5 h and 48 h, respectively [58]. The stability of this protein has not been interrogated, and further study is required to determine whether MTP18 expression is regulated like other mitochondrial dynamic proteins.

## 4. Outer Mitochondrial Membrane Fission and Fusion

OMM fusion is mediated by the mitofusin/fuzzy onions family of large dynamin related GTPases. These proteins are resident in the OMM and stimulate the fusion of these membranes from different mitochondrial compartments. In higher eukaryotes, there are two forms of mitofusin derived from different genes—MFN1 and MFN2. The interactions between mitofusins mediate not only interactions between different mitochondria and their fusion but also interactions between mitochondria and endoplasmic reticulum. The cytoplasmic GTPase domains and heptad repeat and helix bundle domains found in all mitofusin homologs are required for fusion of the OMM. Mitofusin activity is regulated by post-translational ubiquitylation by cytosolic and/or OMM localized E3 ubiquitin ligases that target the mitofusins for degradation by the UPS. Several E3 ubiquitin ligases have been described to ubiquitylate mitofusins including Parkin, MARCH5 (MITOL), HUWE1 (MUL1/MAPL/MULAN/GIDE/MULE/ARF-BP1). MFN2 is ubiquitylated by Parkin after phosphorylation by PINK1 at residue T111 and S242 in the human protein. Although constitutive degradation of mitofusins is required for balance of the mitochondrial network, there are conditions where a change in mitochondrial morphology is required. The stress-activated kinase JNK (c-jun N-terminal kinase) phosphorylates MFN2 on serine 27 after conditions of proteasomal inhibition or inhibition of DNA replication with doxorubicin. Once phosphorylated, Mfn2 is now subject to ubiquitylation by HUWE1 that promotes degradation. The consequences of enhanced MFN2 degradation are mitochondrial fragmentation which acts to inhibit mitochondrial dependent apoptosis [59]. Mitofusin ubiquitylation sites have been identified using quantitative proteomics: human MFN1 has 15 sites, while MFN2 has 14 sites (Table 2) [60]. The function of each of the sites has not been determined, but clues can be obtained from mitofusin homologs in other organisms. The process of Parkin-dependent ubiquitylation of mitofusin is conserved, as the drosophila mitofusin homolog fuzzy Oonions undergoes the same process [61]. The yeast mitofusin homolog, FZO1 (fuzzy onions homolog 1) is ubiquitylated on lysine residues at positions 398 and 464. The lysine residue at position 398 is conserved in yeast and fruit flies while lysine 464 is highly conserved in all eukaryotes, both of which are downstream of the GTPase domain found in all mitofusin homologs. Ubiquitylation is mediated by the CDC34/SCF/MDM30 complex which adds K48 polyubiquitin chains. FZO1 ubiquitylation can be reversed by the action of two deubiqutinase enzymes, UBP2 and UBP12. Ubiquitylation proceeds through initial modification at K464 which is required for K398 to become ubiquitylated. Mutation of K464 to arginine prevents ubiquitylation of FZO1 and also prevents phenotypic complementation of a FZO1 null yeast strain. This indicates that FZO1 ubiquitylation is essential for its activity. FZO1 ubiquitylation at these two different residues is responsible for two different outcomes: K464 is required for UPS-dependent degradation, while K398 is required for correct mitochondrial fusion. Deubiquitylation of these chains occurs by UBP2 in the case of K464 and prevents FZO1 degradation while UBP12 is responsible for K398 deubiquitylation and prevents mitochondrial fusion [62]. The mammalian DUB, USP30, acts on OMM substrates, such as mitofusins that have been ubiquitylated by E3 ligases. Depletion of mitofusins causes mitochondrial fragmentation which is a requirement for mitophagy along with the ubiquitylation OMM proteins that recruit the autophagy machinery to damaged mitochondria [41,63,64,65]. 

In additions to ubiquitylated mitofusin proteins and other OMM proteins acting as a platform to initiate mitophagy, individual ubiquitylated proteins can be extracted from the OMM and targeted to the UPS. The AAA-ATPase VCP/p97 functions to extract ubiquitylated trans-membrane proteins from the endoplasmic reticulum and mitochondria. These extracted proteins are then degraded by the UPS. Along with mitofusins, the anti-apoptotic BCL-2 family member protein MCL-1 is extracted from the OMM for UPS dependent degradation by VCP/p97 once ubiquitylated [66]. 

FZO1 has a half-life that is shorter than the average half-life of all yeast proteins under fermentative growth conditions [47,49]. FZO1 half-life is extended when components of the Cdc34/SCF E2 ubiquitin complex or the mitochondria-associated F-box protein Mdm30 are inactivated, indicating a UPS-dependent mechanism of turnover [67,68]. FZO1 can also be degraded in a proteasome-independent manner through MDM30 (mitochondrial distribution and morphology 30) [69]. In U2OS human osteosarcoma cells MFN2 has a half-life of 3.9 h which is extended in the presence of the proteasome inhibitor lactacystin to 9.4 h and silencing of the ubiquitin E3 ligase HUWE1, indicating the majority of turnover is UPS dependent [59].

In yeast, Ugo1 is a OMM protein that provides a trans-membrane link between FZO1 and MGM1 to form a complex. Absence of UGO1 results in defects in mitochondrial fusion [70,71]. UGO1 is a modified mitochondrial transporter protein that functions during the lipid mixing step of mitochondrial fusion [72]. Turnover of UGO1 protein or ubiqutiylation have not been reported. A human UGO1 homolog, SLC25A46, is also a modified mitochondrial carrier protein that promotes mitochondrial fragmentation when overexpressed. SLC25A46 exists in a complex with MFN1 and MFN2, MFF, and Fis1 in the OMM, and OPA-1 and the cristae remodelling protein mitofilin/FCJ1 in the IMM [33,73]. A mutant of SLC25A46 (L341P), associated with pontocerebellar hypoplasia, is highly unstable compared to wild-type and is degraded in a UPS-dependent mechanism via MARCH5 and HUWE1 [74]. Loss of SLC25A46 stabilizes MFN1 and MFN2 on mitochondria to promote fusion. 

DRP1 is a large GTPase that is responsible for performing the constriction step around the OMM to cause mitochondrial fragmentation. DRP1 resides in the cytoplasm until activated which then causes translocation to mitochondria to perform its function. At mitochondria, DRP1 binds to the receptor Fis1 through MiD49/51. Dynamin family proteins and DNM1 (dynamin-1), the yeast DRP1 homolog, assemble into a helical structure surrounding the site of constriction and upon GTP hydrolysis and conformational change the mitochondria are separated [75]. Post-translational regulation of DRP1 occurs through phosphorylation, nitrosylation, sumoylation and ubiquitylation. Interestingly, MARCH5 and Parkin are also the E3 ubiquitin ligases of DRP1, like the mitofusins. However, ubiquitylation of DRP1 by MARCH5 is not associated with degradation but with translocation of DRP1 to mitochondrial fission sites. On the other hand, Parkin-dependent ubiquitylation of DRP1 occurs through K48 modification and results in increased degradation [76,77]. DRP-1 is also ubiquitylated by APC/C^Cdh1^, an E3 ligase that is activated as cells exit mitosis. DRP1 contains several canonical and non-canonical degradation box motifs. Upon release from synchronized cell cycle arrest DRP1 undergoes cell cycle dependent degradation [78]. The ubiquitylation sites of DRP1 have not been identified as of yet and how ubiquitylation of DRP1 is regulated also needs further investigation. In yeast, DNM1 in yeast has a half-life that is close to the mean of all protein half-lives, indicating this protein is turned over in a similar time-frame as most other proteins in fermentative conditions. 

In yeast, and to a lesser extent in humans, DNM1/DRP-1 is targeted to mitochondria through the OMM resident protein Fis1 [79]. Like other proteins involved in mitochondrial dynamics, Fis1 is ubiquitylated by MARCH5 and Parkin [80]. Fis1 is also ubiquitylated by the E3 ligase, RFN5 (really interesting new gene finger protein 5), which is activated by overexpression of the Parkinson’s disease associated gene, DJ-1. RFN5 translocates to mitochondria to ubiquitylate Fis1 [81]. In humans, Fis1 is also responsible for recruiting TBC1D15 to mitochondria to promote mitophagy. Depletion of TBC1D15 results in fusion of the mitochondrial network indicating a role in mitochondrial fission [82]. However, this is not observed in all cell types [83]. Human TBC1D15 has been identified as a ubiquitylated protein by proteomic approaches on residues K90 and K103 [60]. TBC1D15 protein stability is diminished upon p53 overexpression and nutrient starvation that is dependent lysosomal degradation [84]. In yeast Fis1 requires the action of MDV1 (Gag3, Net2) or CAF4 to recruit DNM1 to mitochondria [85,86,87]. MDV1 (mitochondrial division protein 1) has a half-life of 7.3 h and is ubiquitylated on lysine residue 126 [48,49]. The MDV1 paralog CAF4 has a similar half-life of 7.2 h and there have been no reports of CAF4 ubiquitylation. 

In humans, MiD49 and MiD51 are thought to be required to recruit DRP1 to the OMM. These proteins are resident OMM proteins and form rings and foci with a carboxy terminal domain and may be responsible for binding to inactive DRP1 dimers or inhibiting DRP1 GTPase activity [14,88,89]. MiD49 is a target of MARCH5 resulting in ubiquitylation and UPS dependent degradation [90]. MiD49 was found to be more abundant in MARCH5 depleted cells while the homolog, MiD51 was not changed. MiD49 degradation is induced under conditions of stress, including treatment with the kinase inhibitor, staurosporin, mitochondrial membrane depolarization by FCCP, and inhibition of transcription by actinomycin D. 

MFF is an OMM-localized protein required for mitochondrial fragmentation which may be required for the recruitment of active DRP1 oligomers to the OMM or to activate DRP1 GTPase activity [13,15,88,89,91]. MFF is ubiquitylated by Parkin at a conserved lysine at position 251 after depolarization with mitochondrial uncouplers that results in loss of MFF protein. However, this loss of protein is not dependent on the proteasome and is instead due to mitophagy [92] (Figure 1 and Figure 2).

There are other less well characterized proteins involved in mitochondrial fission and fusion. MSTO1 is a recently described mitochondrial localized protein that promotes fusion [93,94]. MSTO1 has been reported to be modified by ubiquitin in large scale proteomic screens. However, the E3 ubiquitin ligase or regulation and consequence of ubiquitylation have not been determined. GDAP1 is an integral OMM protein that promotes mitochondrial fission and is enriched in the nervous system. Point mutations and truncations of this protein lead to Charcot–Marie tooth disease through impaired mitochondrial fission [95,96]. GDAP1 has been identified as a ubiquitylated protein in large scale proteomic screens. This indicates that depletion of GDAP1 protein would inhibit mitochondrial fragmentation. Gametogenetin-binding protein 1 (GGNBP1) is a mitochondrial fragmentation enhancing protein found in sperm of mice and localized to IMS. 

## 5. Conclusions

The maintenance of a balanced mitochondrial network depends on the action of many different types of proteins that reside in the IMM, IMS, OMM and cytosol. The abundance of these proteins determines whether mitochondrial will undergo fragmentation or fusion. In addition to tissue-specific expression, mitochondrial dynamics proteins do not all possess the same stability, due to different half-lives and ubiquitylation. These post-translational mechanisms of protein expression are therefore subject to complex regulation to ensure that a co-ordinated phenotype of mitochondrial fragmentation or fission is achieved. In addition to the turnover of proteins, proteolytic processing of OPA1/MGM1 in the IMM is a crucial regulator of mitochondrial IMM fusion. Putting these together, there is still much more to uncover regarding the co-ordinated regulation of mitochondrial dynamics of which protein proteolysis and stability are crucial components.

## Figures and Tables

**Table 1:** Yeast mitochondrial dynamics protein half-lives and ubiquitylation properties.

Protein	Half-Life (h) [49]	Half-Life (Mins) [47]	Ubiquitylation Site [48]	E3 Ligase	Deubiquitinase	Function
CAF4	7.2	34	Not reported			
DNM1	10.6	31	Not reported			
FIS1	9.1		Not reported			
FZO1	3	24	79 370 398 464	MDM30 MDM30	UBP12 UBP2	Mitochondrial fusion Degradation
MDV1	7.3	25	126			
MGM1	8.4	Not reported	549			
UGO1	Not reported	Not reported	Not reported			

Note: DNM1: dynamin-1; FIS1: fission 1; FZO1: fuzzy onions homolog 1; MDM30: mitochondrial distribution and morphology 30; MDV1: mitochondrial division protein 1; MGM1: mitochondrial genome maintenance 1.

**Table 2:** Human mitochondrial dynamics protein half-lives and ubiquitylation properties.

Protein	Half-Life (h)	Ubiquitylation Site [60]	E3 Ligase	Deubiquitinase	Function
DRP1		Not reported	MARCH5 Parkin APC/C^Cdh1^		Mitochondrial translocation—non degradative Degradation Degradation after mitosis
Fis1		Not reported	MARCH5 Parkin RFN5		Degradation Degradation Degradation
GDAP1		172 173 188			
MFF		28 251	Parkin		
MFN1		89 91 161 178 181 393 423 467 475 681 746 759			
MFN2	3.9	79 154 158 171 307 316 406 416 420 460 719 720 730 737	Parkin HUWE1	USP30	
MiD49		Not reported	MARCH5		Degradation
MiD51		Not reported			
MSTO1		80 89 105 203 206			
MTP18		Not reported			
OPA1		228 568			
SLC25A46		76			
TBC1D15		90 103			

Note: DRP1: dynamin-related protein; Mff: mitochondrial fission factor; MFN1: Mitofusin1; MiD49: mitochondrial dynamics proteins of 49 kDa; MTP18: mitochondrial protein of 18 kilodaltons; OPA1: optic atrophy 1.

## References

[1] Antico Arciuch V.G., Elguero M.E., Poderoso J.J., Carreras M.C. (2012). Mitochondrial regulation of cell cycle and proliferation. Antioxid. Redox Signal..

[2] Alexeyev M., Shokolenko I., Wilson G., LeDoux S. (2013). The maintenance of mitochondrial DNA integrity—Critical analysis and update. Cold Spring Harb. Perspect. Biol..

[3] Okamoto K., Shaw J.M. (2005). Mitochondrial morphology and dynamics in yeast and multicellular eukaryotes. Annu. Rev. Genet..

[4] Scott I., Youle R.J. (2010). Mitochondrial fission and fusion. Essays Biochem..

[5] Benard G., Karbowski M. (2009). Mitochondrial fusion and division: Regulation and role in cell viability. Semin. Cell Dev. Biol..

[6] Zhang C., Shi Z., Zhang L., Zhou Z., Zheng X., Liu G., Bu G., Fraser P.E., Xu H., Zhang Y.-W. (2016). Appoptosin interacts with mitochondrial outer-membrane fusion proteins and regulates mitochondrial morphology. J. Cell Sci..

[7] Szymański J., Janikiewicz J., Michalska B., Patalas-Krawczyk P., Perrone M., Ziółkowski W., Duszyński J., Pinton P., Dobrzyń A., Więckowski M.R. (2017). Interaction of Mitochondria with the Endoplasmic Reticulum and Plasma Membrane in Calcium Homeostasis, Lipid Trafficking and Mitochondrial Structure. Int. J. Mol. Sci..

[8] Griparic L., van der Wel N.N., Orozco I.J., Peters P.J., van der Bliek A.M. (2004). Loss of the intermembrane space protein Mgm1/OPA1 induces swelling and localized constrictions along the lengths of mitochondria. J. Biol. Chem..

[9] Mishra P., Carelli V., Manfredi G., Chan D.C. (2014). Proteolytic cleavage of Opa1 stimulates mitochondrial inner membrane fusion and couples fusion to oxidative phosphorylation. Cell Metab..

[10] Zhang Y., Chan D.C. (2007). New insights into mitochondrial fusion. FEBS Lett..

[11] Smirnova E., Griparic L., Shurland D.L., van der Bliek A.M. (2001). Dynamin-related protein Drp1 is required for mitochondrial division in mammalian cells. Mol. Biol. Cell.

[12] Mears J.A., Lackner L.L., Fang S., Ingerman E., Nunnari J., Hinshaw J.E. (2011). Conformational changes in Dnm1 support a contractile mechanism for mitochondrial fission. Nat. Struct. Mol. Biol..

[13] Otera H., Wang C., Cleland M.M., Setoguchi K., Yokota S., Youle R.J., Mihara K. (2010). Mff is an essential factor for mitochondrial recruitment of Drp1 during mitochondrial fission in mammalian cells. J. Cell Biol..

[14] Palmer C.S., Osellame L.D., Laine D., Koutsopoulos O.S., Frazier A.E., Ryan M.T. (2011). MiD49 and MiD51, new components of the mitochondrial fission machinery. EMBO Rep..

[15] Losón O.C., Song Z., Chen H., Chan D.C. (2013). Fis1, Mff, MiD49, and MiD51 mediate Drp1 recruitment in mitochondrial fission. Mol. Biol. Cell.

[16] Cribbs J.T., Strack S. (2007). Reversible phosphorylation of Drp1 by cyclic AMP-dependent protein kinase and calcineurin regulates mitochondrial fission and cell death. EMBO Rep..

[17] Saxton W.M., Hollenbeck P.J. (2012). The axonal transport of mitochondria. J. Cell Sci..

[18] Misko A., Jiang S., Wegorzewska I., Milbrandt J., Baloh R.H. (2010). Mitofusin 2 is necessary for transport of axonal mitochondria and interacts with the Miro/Milton complex. J. Neurosci..

[19] Chen H., Chan D.C. (2009). Mitochondrial dynamics—Fusion, fission, movement, and mitophagy—In neurodegenerative diseases. Hum. Mol. Genet..

[20] Kanki T., Klionsky D.J. (2008). Mitophagy in yeast occurs through a selective mechanism. J. Biol. Chem..

[21] Kim I., Rodriguez-Enriquez S., Lemasters J.J. (2007). Selective degradation of mitochondria by mitophagy. Arch. Biochem. Biophys..

[22] Wallace D.C. (2013). Bioenergetics in human evolution and disease: Implications for the origins of biological complexity and the missing genetic variation of common diseases. Philos. Trans. R. Soc. Lond. B Biol. Sci..

[23] Youle R.J., Narendra D.P. (2011). Mechanisms of mitophagy. Nat. Rev. Mol. Cell Biol..

[24] Matsuda N., Sato S., Shiba K., Okatsu K., Saisho K., Gautier C.A., Sou Y.-S., Saiki S., Kawajiri S., Sato F. (2010). PINK1 stabilized by mitochondrial depolarization recruits Parkin to damaged mitochondria and activates latent Parkin for mitophagy. J. Cell Biol..

[25] Narendra D.P., Jin S.M., Tanaka A., Suen D.-F., Gautier C.A., Shen J., Cookson M.R., Youle R.J. (2010). PINK1 is selectively stabilized on impaired mitochondria to activate Parkin. PLoS Biol..

[26] Park J., Lee S.B., Lee S., Kim Y., Song S., Kim S., Bae E., Kim J., Shong M., Kim J.-M. (2006). Mitochondrial dysfunction in Drosophila PINK1 mutants is complemented by parkin. Nature.

[27] Greene A.W., Grenier K., Aguileta M.A., Muise S., Farazifard R., Haque M.E., McBride H.M., Park D.S., Fon E.A. (2012). Mitochondrial processing peptidase regulates PINK1 processing, import and Parkin recruitment. EMBO Rep..

[28] Ni H.-M., Williams J.A., Ding W.-X. (2015). Mitochondrial dynamics and mitochondrial quality control. Redox Biol..

[29] Liesa M., Palacín M., Zorzano A. (2009). Mitochondrial dynamics in mammalian health and disease. Physiol. Rev..

[30] Fahrner J.A., Liu R., Perry M.S., Klein J., Chan D.C. (2016). A novel de novo dominant negative mutation in DNM1L impairs mitochondrial fission and presents as childhood epileptic encephalopathy. Am. J. Med. Genet. A.

[31] Sheffer R., Douiev L., Edvardson S., Shaag A., Tamimi K., Soiferman D., Meiner V., Saada A. (2016). Postnatal microcephaly and pain insensitivity due to a de novo heterozygous DNM1L mutation causing impaired mitochondrial fission and function. Am. J. Med. Genet. A.

[32] Pickrell A.M., Youle R.J. (2015). The roles of PINK1, parkin, and mitochondrial fidelity in Parkinson’s disease. Neuron.

[33] Abrams A.J., Hufnagel R.B., Rebelo A., Zanna C., Patel N., Gonzalez M.A., Campeanu I.J., Griffin L.B., Groenewald S., Strickland A.V. (2015). Mutations in SLC25A46, encoding a UGO1-like protein, cause an optic atrophy spectrum disorder. Nat. Genet..

[34] Züchner S., De Jonghe P., Jordanova A., Claeys K.G., Guergueltcheva V., Cherninkova S., Hamilton S.R., Van Stavern G., Krajewski K.M., Stajich J. (2006). Axonal neuropathy with optic atrophy is caused by mutations in mitofusin 2. Ann. Neurol..

[35] Froyen G., Corbett M., Vandewalle J., Jarvela I., Lawrence O., Meldrum C., Bauters M., Govaerts K., Vandeleur L., Van Esch H. (2008). Submicroscopic duplications of the hydroxysteroid dehydrogenase HSD17B10 and the E3 ubiquitin ligase HUWE1 are associated with mental retardation. Am. J. Hum. Genet..

[36] Ong S.-B., Subrayan S., Lim S.Y., Yellon D.M., Davidson S.M., Hausenloy D.J. (2010). Inhibiting mitochondrial fission protects the heart against ischemia/reperfusion injury. Circulation.

[37] Yu T., Sheu S.-S., Robotham J.L., Yoon Y. (2008). Mitochondrial fission mediates high glucose-induced cell death through elevated production of reactive oxygen species. Cardiovasc. Res..

[38] Xu S., Wang P., Zhang H., Gong G., Gutierrez Cortes N., Zhu W., Yoon Y., Tian R., Wang W. (2016). CaMKII induces permeability transition through Drp1 phosphorylation during chronic β-AR stimulation. Nat. Commun..

[39] Kong D., Xu L., Yu Y., Zhu W., Andrews D.W., Yoon Y., Kuo T.H. (2005). Regulation of Ca^2+^-induced permeability transition by Bcl-2 is antagonized by Drp1 and hFis1. Mol. Cell Biochem..

[40] Matsumoto M., Hatakeyama S., Oyamada K., Oda Y., Nishimura T., Nakayama K.I. (2005). Large-scale analysis of the human ubiquitin-related proteome. Proteomics.

[41] Cunningham C.N., Baughman J.M., Phu L., Tea J.S., Yu C., Coons M., Kirkpatrick D.S., Bingol B., Corn J.E. (2015). USP30 and parkin homeostatically regulate atypical ubiquitin chains on mitochondria. Nat. Cell Biol..

[42] Altmann K., Westermann B. (2005). Role of essential genes in mitochondrial morphogenesis in Saccharomyces cerevisiae. Mol. Biol. Cell.

[43] Dantuma N.P., Bott L.C. (2014). The ubiquitin-proteasome system in neurodegenerative diseases: Precipitating factor, yet part of the solution. Front. Mol. Neurosci..

[44] Bragoszewski P., Turek M., Chacinska A. (2017). Control of mitochondrial biogenesis and function by the ubiquitin-proteasome system. Open Biol..

[45] Sugiura A., McLelland G.-L., Fon E.A., McBride H.M. (2014). A new pathway for mitochondrial quality control: Mitochondrial-derived vesicles. EMBO J..

[46] Eden E., Geva-Zatorsky N., Issaeva I., Cohen A., Dekel E., Danon T., Cohen L., Mayo A., Alon U. (2011). Proteome half-life dynamics in living human cells. Science.

[47] Belle A., Tanay A., Bitincka L., Shamir R., O’Shea E.K. (2006). Quantification of protein half-lives in the budding yeast proteome. Proc. Natl. Acad. Sci. USA.

[48] Swaney D.L., Beltrao P., Starita L., Guo A., Rush J., Fields S., Krogan N.J., Villén J. (2013). Global analysis of phosphorylation and ubiquitylation cross-talk in protein degradation. Nat. Methods.

[49] Christiano R., Nagaraj N., Fröhlich F., Walther T.C. (2014). Global proteome turnover analyses of the Yeasts *S. cerevisiae* and *S. pombe*. Cell Rep..

[50] Ishihara N., Fujita Y., Oka T., Mihara K. (2006). Regulation of mitochondrial morphology through proteolytic cleavage of OPA1. EMBO J..

[51] Baker M.J., Lampe P.A., Stojanovski D., Korwitz A., Anand R., Tatsuta T., Langer T. (2014). Stress-induced OMA1 activation and autocatalytic turnover regulate OPA1-dependent mitochondrial dynamics. EMBO J..

[52] Head B., Griparic L., Amiri M., Gandre-Babbe S., van der Bliek A.M. (2009). Inducible proteolytic inactivation of OPA1 mediated by the OMA1 protease in mammalian cells. J. Cell Biol..

[53] Del Dotto V., Mishra P., Vidoni S., Fogazza M., Maresca A., Caporali L., McCaffery J.M., Cappelletti M., Baruffini E., Lenaers G. (2017). OPA1 isoforms in the hierarchical organization of mitochondrial functions. Cell Rep..

[54] Bohovych I., Donaldson G., Christianson S., Zahayko N., Khalimonchuk O. (2014). Stress-triggered activation of the metalloprotease Oma1 involves its C-terminal region and is important for mitochondrial stress protection in yeast. J. Biol. Chem..

[55] Herlan M., Vogel F., Bornhovd C., Neupert W., Reichert A.S. (2003). Processing of Mgm1 by the rhomboid-type protease Pcp1 is required for maintenance of mitochondrial morphology and of mitochondrial DNA. J. Biol. Chem..

[56] Chan E.Y.L., McQuibban G.A. (2012). Phosphatidylserine decarboxylase 1 (Psd1) promotes mitochondrial fusion by regulating the biophysical properties of the mitochondrial membrane and alternative topogenesis of mitochondrial genome maintenance protein 1 (Mgm1). J. Biol. Chem..

[57] Tondera D., Czauderna F., Paulick K., Schwarzer R., Kaufmann J., Santel A. (2005). The mitochondrial protein MTP18 contributes to mitochondrial fission in mammalian cells. J. Cell Sci..

[58] Tondera D., Santel A., Schwarzer R., Dames S., Giese K., Klippel A., Kaufmann J. (2004). Knockdown of MTP18, a novel phosphatidylinositol 3-kinase-dependent protein, affects mitochondrial morphology and induces apoptosis. J. Biol. Chem..

[59] Leboucher G.P., Tsai Y.C., Yang M., Shaw K.C., Zhou M., Veenstra T.D., Glickman M.H., Weissman A.M. (2012). Stress-induced phosphorylation and proteasomal degradation of mitofusin 2 facilitates mitochondrial fragmentation and apoptosis. Mol. Cell.

[60] Kim W., Bennett E.J., Huttlin E.L., Guo A., Li J., Possemato A., Sowa M.E., Rad R., Rush J., Comb M.J. (2011). Systematic and quantitative assessment of the ubiquitin-modified proteome. Mol. Cell.

[61] Poole A.C., Thomas R.E., Yu S., Vincow E.S., Pallanck L. (2010). The mitochondrial fusion-promoting factor mitofusin is a substrate of the PINK1/parkin pathway. PLoS ONE.

[62] Anton F., Dittmar G., Langer T., Escobar-Henriques M. (2013). Two deubiquitylases act on mitofusin and regulate mitochondrial fusion along independent pathways. Mol. Cell.

[63] Bingol B., Tea J.S., Phu L., Reichelt M., Bakalarski C.E., Song Q., Foreman O., Kirkpatrick D.S., Sheng M. (2014). The mitochondrial deubiquitinase USP30 opposes parkin-mediated mitophagy. Nature.

[64] Nakamura N., Hirose S. (2008). Regulation of mitochondrial morphology by USP30, a deubiquitinating enzyme present in the mitochondrial outer membrane. Mol. Biol. Cell.

[65] Liang J.-R., Martinez A., Lane J.D., Mayor U., Clague M.J., Urbé S. (2015). USP30 deubiquitylates mitochondrial Parkin substrates and restricts apoptotic cell death. EMBO Rep..

[66] Xu S., Peng G., Wang Y., Fang S., Karbowski M. (2011). The AAA-ATPase p97 is essential for outer mitochondrial membrane protein turnover. Mol. Biol. Cell.

[67] Cohen M.M.J., Leboucher G.P., Livnat-Levanon N., Glickman M.H., Weissman A.M. (2008). Ubiquitin-proteasome-dependent degradation of a mitofusin, a critical regulator of mitochondrial fusion. Mol. Biol. Cell.

[68] Fritz S., Weinbach N., Westermann B. (2003). Mdm30 is an F-box protein required for maintenance of fusion-competent mitochondria in yeast. Mol. Biol. Cell.

[69] Escobar-Henriques M., Westermann B., Langer T. (2006). Regulation of mitochondrial fusion by the F-box protein Mdm30 involves proteasome-independent turnover of Fzo1. J. Cell Biol..

[70] Sesaki H., Jensen R.E. (2001). UGO1 encodes an outer membrane protein required for mitochondrial fusion. J. Cell Biol..

[71] Sesaki H., Jensen R.E. (2004). Ugo1p links the Fzo1p and Mgm1p GTPases for mitochondrial fusion. J. Biol. Chem..

[72] Hoppins S., Horner J., Song C., McCaffery J.M., Nunnari J. (2009). Mitochondrial outer and inner membrane fusion requires a modified carrier protein. J. Cell Biol..

[73] Janer A., Prudent J., Paupe V., Fahiminiya S., Majewski J., Sgarioto N., Des Rosiers C., Forest A., Lin Z.-Y., Gingras A.-C. (2016). SLC25A46 is required for mitochondrial lipid homeostasis and cristae maintenance and is responsible for Leigh syndrome. EMBO Mol. Med..

[74] Steffen J., Vashisht A.A., Wan J., Jen J.C., Claypool S.M., Wohlschlegel J.A., Koehler C.M. (2017). Rapid degradation of mutant SLC25A46 by the ubiquitin-proteasome system results in MFN1/2-mediated hyperfusion of mitochondria. Mol. Biol. Cell.

[75] Ingerman E., Perkins E.M., Marino M., Mears J.A., McCaffery J.M., Hinshaw J.E., Nunnari J. (2005). Dnm1 forms spirals that are structurally tailored to fit mitochondria. J. Cell Biol..

[76] Nakamura N., Kimura Y., Tokuda M., Honda S., Hirose S. (2006). MARCH-V is a novel mitofusin 2- and Drp1-binding protein able to change mitochondrial morphology. EMBO Rep..

[77] Wang H., Song P., Du L., Tian W., Yue W., Liu M., Li D., Wang B., Zhu Y., Cao C. (2011). Parkin ubiquitinates Drp1 for proteasome-dependent degradation: Implication of dysregulated mitochondrial dynamics in Parkinson disease. J. Biol. Chem..

[78] Horn S.R., Thomenius M.J., Johnson E.S., Freel C.D., Wu J.Q., Coloff J.L., Yang C.-S., Tang W., An J., Ilkayeva O.R. (2011). Regulation of mitochondrial morphology by APC/CCdh1-mediated control of Drp1 stability. Mol. Biol. Cell.

[79] Yoon Y., Krueger E.W., Oswald B.J., McNiven M.A. (2003). The mitochondrial protein hFis1 regulates mitochondrial fission in mammalian cells through an interaction with the dynamin-like protein DLP1. Mol. Cell Biol..

[80] Yonashiro R., Ishido S., Kyo S., Fukuda T., Goto E., Matsuki Y., Ohmura-Hoshino M., Sada K., Hotta H., Yamamura H. (2006). A novel mitochondrial ubiquitin ligase plays a critical role in mitochondrial dynamics. EMBO J..

[81] Zhang Q., Wu J., Wu R., Ma J., Du G., Jiao R., Tian Y., Zheng Z., Yuan Z. (2012). DJ-1 promotes the proteasomal degradation of Fis1: Implications of DJ-1 in neuronal protection. Biochem. J..

[82] Onoue K., Jofuku A., Ban-Ishihara R., Ishihara T., Maeda M., Koshiba T., Itoh T., Fukuda M., Otera H., Oka T. (2013). Fis1 acts as a mitochondrial recruitment factor for TBC1D15 that is involved in regulation of mitochondrial morphology. J. Cell Sci..

[83] Yamano K., Fogel A.I., Wang C., van der Bliek A.M., Youle R.J. (2014). Mitochondrial Rab GAPs govern autophagosome biogenesis during mitophagy. eLife.

[84] Feldman D.E., Chen C., Punj V., Machida K. (2013). The TBC1D15 oncoprotein controls stem cell self-renewal through destabilization of the Numb-p53 complex. PLoS ONE.

[85] Tieu Q., Okreglak V., Naylor K., Nunnari J. (2002). The WD repeat protein, Mdv1p, functions as a molecular adaptor by interacting with Dnm1p and Fis1p during mitochondrial fission. J. Cell Biol..

[86] Fekkes P., Shepard K.A., Yaffe M.P. (2000). Gag3p, an outer membrane protein required for fission of mitochondrial tubules. J. Cell Biol..

[87] Cerveny K.L., McCaffery J.M., Jensen R.E. (2001). Division of mitochondria requires a novel DMN1-interacting protein, Net2p. Mol. Biol. Cell.

[88] Liu R., Chan D.C. (2015). The mitochondrial fission receptor Mff selectively recruits oligomerized Drp1. Mol. Biol. Cell.

[89] Osellame L.D., Singh A.P., Stroud D.A., Palmer C.S., Stojanovski D., Ramachandran R., Ryan M.T. (2016). Cooperative and independent roles of the Drp1 adaptors Mff, MiD49 and MiD51 in mitochondrial fission. J. Cell Sci..

[90] Xu S., Cherok E., Das S., Li S., Roelofs B.A., Ge S.X., Polster B.M., Boyman L., Lederer W.J., Wang C. (2016). Mitochondrial E3 ubiquitin ligase MARCH5 controls mitochondrial fission and cell sensitivity to stress-induced apoptosis through regulation of MiD49 protein. Mol. Biol. Cell.

[91] Clinton R.W., Francy C.A., Ramachandran R., Qi X., Mears J.A. (2016). Dynamin-related Protein 1 Oligomerization in Solution Impairs Functional Interactions with Membrane-anchored Mitochondrial Fission Factor. J. Biol. Chem..

[92] Gao J., Qin S., Jiang C. (2015). Parkin-induced ubiquitination of Mff promotes its association with p62/SQSTM1 during mitochondrial depolarization. Acta Biochim. Biophys. Sin..

[93] Gal A., Balicza P., Weaver D., Naghdi S., Joseph S.K., Várnai P., Gyuris T., Horváth A., Nagy L., Seifert E.L. (2017). MSTO1 is a cytoplasmic pro-mitochondrial fusion protein, whose mutation induces myopathy and ataxia in humans. EMBO Mol. Med..

[94] Kimura M., Okano Y. (2007). Human Misato regulates mitochondrial distribution and morphology. Exp. Cell Res..

[95] Wagner K.M., Rüegg M., Niemann A., Suter U. (2009). Targeting and function of the mitochondrial fission factor GDAP1 are dependent on its tail-anchor. PLoS ONE.

[96] Niemann A., Wagner K.M., Ruegg M., Suter U. (2009). GDAP1 mutations differ in their effects on mitochondrial dynamics and apoptosis depending on the mode of inheritance. Neurobiol. Dis..

